# FC-EODR: Immersive Humanoid Dual-Arm Dexterous Explosive Ordnance Disposal Robot

**DOI:** 10.3390/biomimetics8010067

**Published:** 2023-02-06

**Authors:** Zhihong Jiang, Yifan Ma, Xiaolei Cao, Minghui Shen, Chunlong Yin, Hongyang Liu, Junhan Cui, Zeyuan Sun, Xiao Huang, Hui Li

**Affiliations:** The School of Mechatronical Engineering, Beijing Institute of Technology, Beijing 100081, China

**Keywords:** explosive ordnance disposal robot, cooperative manipulator, teleoperation, tool design, tracked robot

## Abstract

In this study, we proposes a humanoid dual-arm explosive ordnance disposal (EOD) robot design. First, a seven-degree-of-freedom high-performance collaborative and flexible manipulator is developed, aiming at the transfer and dexterous operation of dangerous objects in EOD tasks. Furthermore, an immersive operated humanoid dual-arm dexterous explosive disposal robot (FC-EODR) is designed, which has a high passability to complex terrains such as low walls, slope roads, and stairs. It can remotely detect, manipulate, and remove explosives in dangerous environments through immersive velocity teleoperation. In addition, an autonomous tool-changing system is constructed, which enables the robot to flexibly switch between different tasks. The effectiveness of the FC-EODR is finally verified through a series of experiments, including the platform performance test, manipulator load test, teleoperated wire trimming, and screw-screwing experiments. This letter provides the technical foundation for robots to replace humans in EOD tasks and emergency situations.

## 1. Introduction

Explosive ordnance disposal (EOD) robots play an important role in countering terrorist attacks and handling hazardous environments. During the manual EOD, an operator removes found suspicious objects after checking them, usually by in situ destruction. The explosives that cannot be defused need to be transferred to a safe area for detonation or destruction by methods such as wire cutting and detonator removal. The EOD robots are specifically designed to perform EOD tasks.

Many EOD robots have been shown to be effective in EOD tasks [1]. The typical configuration of EOD robots is a tracked chassis and a single manipulator configuration, such as FLIR’s PackBot robot [2], NIDES’s TELEROB [3], and Jingpin’ JP-REOD400 [4]. However, there are performances that can be improved in the application of EOD robot. For a typical single-arm and crawler configuration, the manipulator usually has large load but low operation accuracy and large backlash as the joint actuator uses worm-gear reducer. This may require operators to estimate the robot state through extensive training and significantly increases the difficulty of handling the explosives and the completion time of operational tasks. In the manipulation task, the humanlike dual-arm configuration can make the operators apply more bimanual experience to the manipulation task to improve the flexibility [5]. Many robots with dual-arm configuration, such as BIT-DMR [6] and HRP-5P [7], has been proved to be efficient in emergency rescue scene. In EOD tasks, RE2’ Highly Dexterous Manipulation System (HDMS) uses imitative controllers and achieves humanlike agility in dextrous manipulation [8]. Dual-arm robots with force-controlled collaborative manipulators, such as DLR’s Justin robot [9] with lightweight robotics arms [10,11], can utilize the fine force control manipulation capability of the cooperative manipulators to improve the overall robot dexterous manipulation performance. The force-controlled cooperative manipulators have not yet been applied in EOD robots as far as we know.

The EOD tasks are typically highly dependent on tools. Special tools perform better than general tools in specific tasks, which usually requires EOD robots to have certain adaptability to multiple tools. However, the method of manual tool replacement can cause a problem in scenarios in which robots and operators need to be far apart. Some designs adopt a combination of multiple tools [12]. The ability to replace tools autonomously is necessary for the EOD robot.

The user interface design of an EOD robot is also an important topic worthy of study. In an EOD scene, an operator is usually in a state of high mental stress due to the need to constantly pay attention to the state of operating tools and suspicious objects. In the current EOD robots, joysticks and buttons are generally used for control in manipulator joint space. Because it is not intuitive, an operator needs much learning and training [13], and a large cognitive load will be imposed. Teleoperation is still the main operation method of EOD robots. The improvement in the teleoperation workflow combined with environmental perception has also been a research direction to improve operational efficiency. The environment can affect the accuracy and speed of operation [14]; hence, combining the perception data of an environment and a human decision can improve the object recognition ability of a human operator and the operation ability of a robot [15]. Based on teleoperation, there have been many studies on the partial autonomous intelligence of manipulator operation, such as manipulator path planning [16], optimization of redundant manipulators [17], and the use of reinforcement learning method [18], which are used to reduce the cognitive pressure on an operator. The hand-eye calibration problem when using vision feedback was well handled [19]. There are also some work on autonomous intelligence for mobile platforms [20]. Autonomous attempts have received positive feedback in interviews with EOD robot operators [21]. The contribution of the article can be summarized as follows:

1. An immersive teleoperated humanoid dual-arm dexterous EOD robot named the FC-EODR is designed, in which mobile platform, high-position accuracy and force-controlled manipulator, and autoreplacement tool can effectively improve the passability of EOD process, high-precision and dexterous operation ability, stable transfer ability and multitool operation ability.
2. A new immersive teleoperation system is constructed, and a semiautomatic compliant teleoperation method based on point cloud interaction is proposed, which can further improve the success rate of complex EOD operations. It is verified in cutting wires, screwing, and cutting cardboard tasks.

## 2. Robot Mechanical System Design

**Figure 1:**
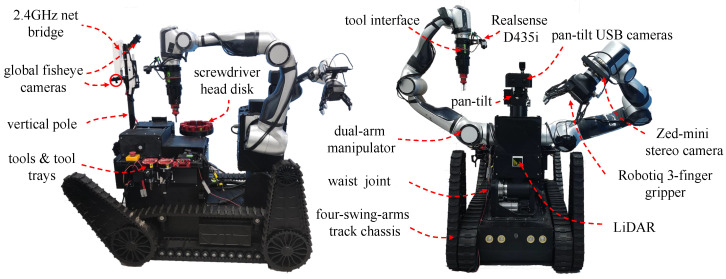
The overall appearance of the proposed robot.

The FC-EODR is mainly composed of a chassis, two manipulators, an autoreplacement tool system, and a perception system. The overall structure of the proposed FC-EODR is shown in Figure 1. The robot is driven by electric power. The weight of the whole machine is 350 kg, and it adopts a four-swing arm crawler chassis. The dual-arm part has a humanoid configuration with two collaborative redundant manipulators. One Robotiq 3-Finger Adaptive Gripper [22] is installed on the left manipulator, and one tool interface is installed on the right manipulator.

### 2.1. High-Passability Mobile Platform Design

In the urban scenes that usually appear in an EOD environment, there can be complex surfaces, such as steps, low walls, uphill, sand, and gravel [23]. Among them, the appearance of steps can significantly reduce the passability of a wheeled chassis. In contrast, due to the long ground contact line, the crawler type can pass through the stepped road well. In the low-wall scenario, a chassis needs to cross vertical obstacles of about 0.5 m, and a typical crawler chassis is limited to its front climbing height. The front swing arms can rotate to increase climbing height, and the rear swing arm can rotate to stabilize the body and reduce the impact during the touchdown process of low wall crossing task. Swing arms can be flattened to increase length of ground contact line when passing through the steps.

**Figure 2:**
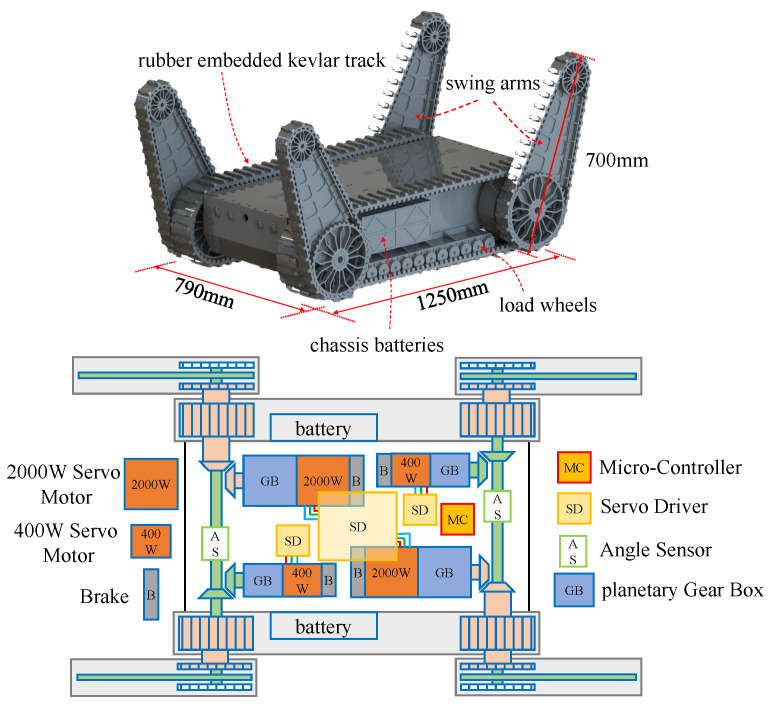
Mechanical dimensions and internal layout of the mobile platform.

The FC-EODR adopts a four-swing arm crawler chassis, where two main crawlers drive platform, and the four swing crawlers rotate to realize a vertical movement of the chassis to complete obstacle crossing smoothly. The chassis structure and its power distribution are shown in Figure 2. The main body of the four-swing arm crawler chassis is an aluminum casing. The torque output shaft is placed at the symmetrical front and rear positions of the chassis. The output shaft has a coaxial dual-output design. The external shaft transfers power to the main track, which drives the driven wheel to synchronize the speed of the main track and the swing arm track on one side. The direction of the car body is adjusted through the differential speed. The left and right swing arms are fixed together through the internal shaft, resulting in the angle of the two front or two rear swing arms to be the same. The drive motor, reducer, driver, and microcontroller are placed inside the casing. To balance the center of gravity of a vehicle, the motors are placed symmetrically on the front and rear sides. The main track reducer and the swing arm track reducer use the planetary reducer, and bevel gears are employed to turn the power to 90° for output. Between the main track and the casing, tensioner wheels and road wheels are installed.

The platform can achieve speeds of at least 1.5 m/s, obstacle crossing height of 0.5 m, and the cross-ditch width of at least 0.4 m. Its climbing angle is equal to 35° (can climb stairs). The mobile platform can realize zero radius steering and reliable movements both indoors and outdoors and on complex roads situation. The platform is designed to carry a load of 120 kg. At the same time, the swing arm motor can support its own weight and load to complete the task of overcoming obstacles.

### 2.2. High-Performance Force-Controlled Collaborative Manipulator Design

Considering the dangers of an EOD scene and the requirements for precise operation, especially the requirements for the stability and acceleration limit of the manipulator end-effector in the transferring task, the position control accuracy and force control performance of the end-effector of a manipulator are the main design goals considered in this study.

**Figure 3:**
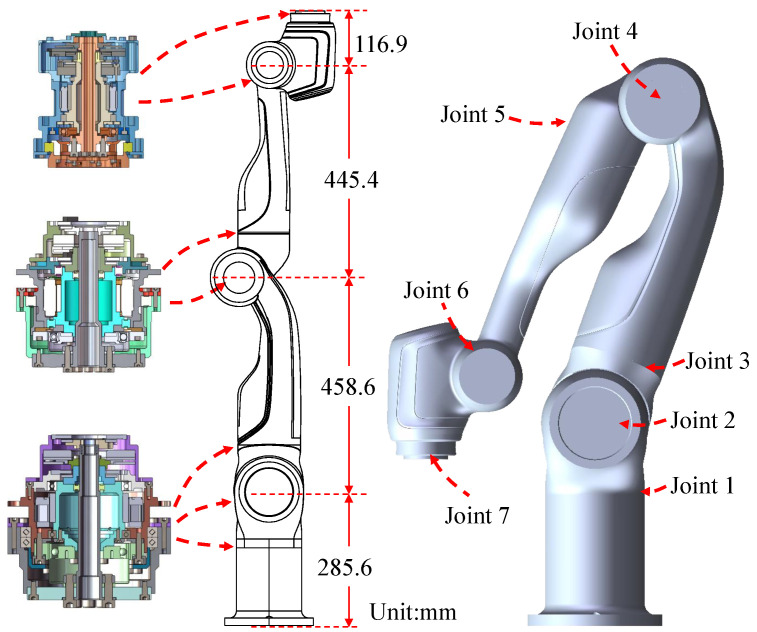
Structural form of the manipulator.

The manipulator of FC-EODR has a humanoid configuration, where a single arm has seven degrees of freedom (Dof), three shoulder joints, three wrist joints, and one elbow joint. Aiming at the general form of target objects for EOD and small suspicious explosives, the FC-EODR manipulator has an operational load capacity of 10 kg, which can be lifted and operated stably. In an EOD operation scene, the workspace of human arms can meet most needs. The FC-EODR adopts a double-arm design, and the arm workspace of the manipulator is slightly larger than the human arm workspace. This manipulator has a working radius of 914 mm which meets the needs of workspace and is enough to fetch tools mounted behind the torso. The shape of the manipulator are shown in Figure 3. The performance parameters are shown in Table 1.

Aiming at the problem of end-effector positioning accuracy and stability, the proposed robot uses a harmonic reducer as a joint reducer due to its characteristics of small backlash and large reduction ratio. The links are designed with a cylinder and have a large safety factor to ensure the rigidity of the connecting rod and improve the positioning accuracy. The design weight of the single arm is 26 kg, and the repeat positioning accuracy of the end is ±0.03 mm. To meet the acceleration and stability requirements of a stable transfer task, a force control scheme is used for the rotating joint of the manipulator, and a torque sensor is installed on the joint output shaft for joint force perception. The control scheme mainly refers to the design of DLR LWR [10].

### 2.3. Automatic Tool Replacement Interface Design

**Figure 4:**
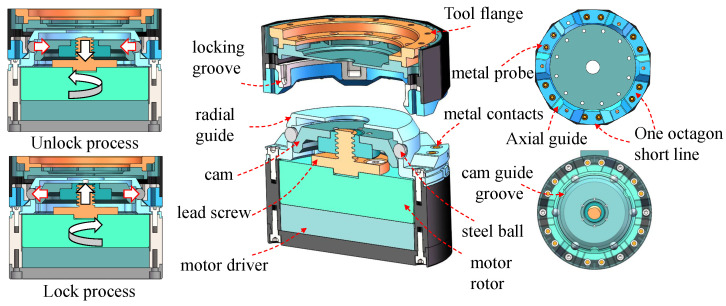
The hardware design of the tool interface module.

To achieve the function of automatic replacement of various tools, this study designs a lightweight electrical connection tool replacement interface. This interface uses a screw-cam to construct a mechanical interface and a spring probe to form a circuit interface. A variety of special tools and tool seats are designed. The autoreplacement interface is divided into two parts, namely the interface module and the tool module. The interface module is always fixed at the end of the manipulator during operation, and the tool module carries different tools. The interface module adopts a single-drive multiple-cam step-by-step connection method and uses a cam stroke differential step to realize the mechanical–electrical connection. The structure of the interface module is shown in Figure 4.

The locking mechanism adopts a steel ball mechanism, and the steel ball is fixed to the side wall by the cam. As shown in the left part of Figure 4, when the locking operation is in progress, the motor drives the lead screw to rotate, thereby guiding the cam to move in the axial direction, thereby pushing the steel ball to move in the radial direction, and then the steel ball touches the locking groove to securely connect the tool module. The unlock operation is performed in reverse. The tapered surface guide is adopted in both radial and axial directions to meet the large-tolerance requirements. This mechanical connection method has the benefits of simultaneous positioning and locking. The output tension can reach 550 N, the load torque is at least 100 Nm, and the load bending moment is at least 100 Nm, so the robot can adapt to more tools and large loads. The steel ball locking has the characteristic of high positioning accuracy, which is a prerequisite for precise operation. The spatial position accuracy is 0.1 mm, and the attitude accuracy is 0.01°. The connection occlusion is a regular octagonal structure, while one gap slope is different to prevent docking errors. The manipulator needs to guide the interface module to the appropriate direction to prevent docking failure (see tool replacement process details in Supplementary Video). In the tool system, the interface module provides a 24-V 4-A (maximum) power supply and the RS-485 communication bus for maximum compatibility.

**Figure 5:**
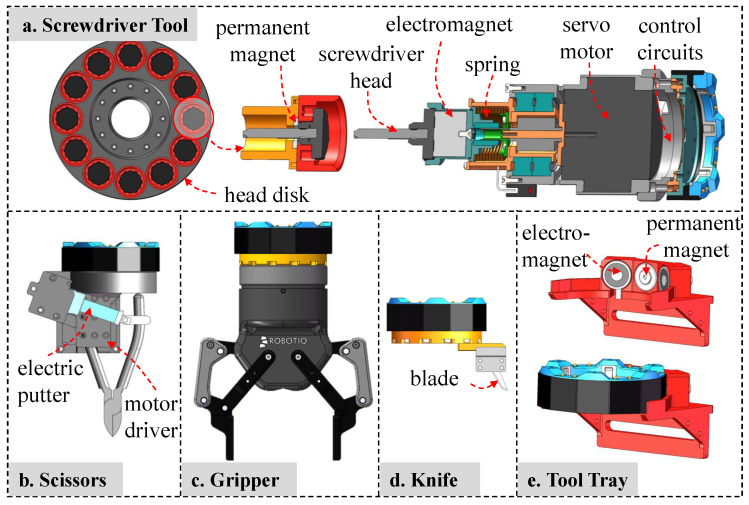
The hardware design of the working tool module.

The working tools are designed and improved in this study, including blades, electric wire cutters, electric grippers, and electric screwdrivers, as shown in Figure 5. The electric screwdriver, which is presented in Figure 5a, is designed to replace the screwdriver head considering various types of screws that need to be operated. The screwdriver tool is designed with a reduced brushless motor, and a linear spring is added to buffer the impact power during the butt screw procedure. An electromagnet is installed at the end of the screwdriver to attract and disconnect the heads. The zero-position of a screwdriver is detected by the photoelectric switch. Twelve screwdriver heads are placed in a circular shape, which is convenient for the manipulator to find the corresponding head during the tool replacement process. The electric wire cutter, as shown in Figure 5b, uses an electric push rod to push and pull the handle of pliers to complete the wire trimming operation. The electric gripper, as shown in Figure 5c, adopts the 2F-85 Adaptive Grippers [24] manufactured by Robotiq company. The knife shown in Figure 5d carries a blade. It is also convenient to carry other special equipment for an EOD task, such as portable X-ray machines and explosives destroyers, which can be installed and placed on the robot through the replacement interface, and taken out for use when needed. An electromagnet magnetic attraction tray is used to ensure the tools will not fall off, as shown in Figure 5e. A 24-V power supply on the probe and serial port signals are processed to drive the motor movement and special actions such as homing.

### 2.4. Perception System Design

The FC-EODR uses five different cameras and LiDAR to achieve the multiform perception of an environment. First, for the manipulator operation task, a hand-eye camera is used as the main observation method. For the left manipulator with 3-Finger adaptive gripper, a binocular camera zed-mini is used to perceive the depth. An RGB-D camera is used on the autoreplacement interface side to achieve the point cloud collection for a local environment. Two network cameras used to collect images are located on the vertical pole at the tail of the FC-EODR. One of them is used to observe the overall perspective of the robot’s operation, and the other is used to observe the rear perspective of the robot. Both cameras use fisheye lenses with a field of view of 185°, which can accurately observe obstacles in an environment and the state of the robot. Two USB cameras are placed on the two-axis gimbal of the robot head. One with a fisheye lens provides a wide range of observation and the other with a telephoto lens provides high-resolution details. LiDAR is used for the environment mapping and automatic navigation applications.

## 3. Robot Control System

EOD robot operations require precise operation, stable transfer, and use of several tools. Real-time teleoperation and semiautomatic control are two main method to handle these needs. We propose a teleoperation method for specific tool scenarios that combines point cloud interaction and manipulator force control to shorten operation time, reduce operation uncertainty, and improve the operation success rate.

### 3.1. Teleoperation Based on Point Cloud Perception

The main features of the precise operation tasks are a narrow operating range, small operating targets, and high precision requirements. The operating time requirements are not strict, but the requirement for a high operating success rate is important.

In view of such characteristics, a teleoperation method of controlling the velocity of the manipulator end-effector in the Cartesian space is adopted, which cooperates with the hand-eye camera image, point cloud perception, and force controller to complete the task of teleoperation. The entire workflow combines the advantages of a human operator’s strong ability and sufficient experience to understand the operating targets, the advantages of dynamic adjustment based on force control and positioning accuracy of a manipulator, and the advantages of the local accuracy of vision sensors; then, the robot’s ability to detect and operate can be improved.

**Figure 6:**
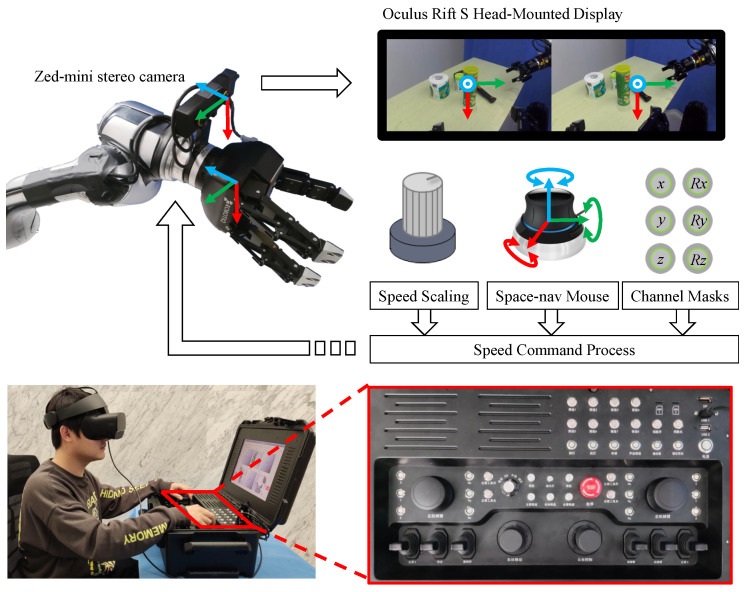
Schematic diagram of the immersive teleoperation operation.

An operator holds a six-dimensional (6D) spece-nav mouse in his or her left and right hands to control the left and right manipulators, respectively. A head-mounted display shows images from the hand-eye camera. The immersive display guide an operator to focus on an image and improve the attention to the key information of a dangerous situation. After pushing the 6D mouse, data are subjected to the dead zone compensation, speed scale mapping, and channel masking, and then the processed value is mapped to the velocity command of the manipulator end-effector in end-effector frame. The joystick with six degrees of freedom controls the six degrees of freedom of the manipulator end-effector velocity. An intuitive mapping is established, which aligns the image coordinate system and the handheld 6D mouse coordinate system as shown in Figure 6. All Dof of manipulator end-effectors are controlled without much thinking when the operator imagines himself working by holding the 6D mouse as the camera (or the end-effector) and understands that the camera is fixed with the end-effector. The schematic diagram of the immersive teleoperation operation is shown in Figure 6. For manipulator joint angle constraints, joint velocity is optimized in teleoperation process using redundant degrees of freedom of manipulator [25] as shown in (1),

(1) $$\begin{array}{c} \begin{array}{cc} \dot{q} & =J^{†}v_{d}+\left(I_{n}-J^{†}J\right)k_{0}{\left(\frac{\partial w(q)}{\partial q}\right)}^{T}\\ w(q) & =-\frac{1}{2n}\sum_{i=1}^{n}{\left(\frac{q_{i}-{\bar{q}}_{i}}{q_{iu}-q_{il}}\right)}^{2} \end{array} \end{array}$$

where q is the joint position and $\dot{q}$ is the joint velocity. J is the Jacobian of the manipulator and $J^{†}$ is its pseudoinverse matrix. $v_{d}$ is the desired end-effector velocity from the user command. $I_{n}$ is a n-dimensional identity matrix and n=7 here in this article. $q_{i}$ is the joint position of joint *i*, $q_{iu}$ is the upper limit of joint *i*, $q_{il}$ is the lower limit of joint *i*, and ${\bar{q}}_{i}$ is the average of upper and lower limits of joint *i*. $k_{0}$ is a positive definite diagonal matrix used to adjust the magnitude of the null-space joint velocity.

When completing tasks with environmental constraints, such as manual alignment in screwing tasks, and maintaining contact in cutting cardboard tasks, the manual teleoperation makes it difficult, time-consuming, and inaccurate. A visual assistance system is designed. The environment is reconstructed, and a user simply clicks to select and supervise the execution to guide the manipulator to reach the fine operation area while meeting the preliminary operation constraints.

**Figure 7:**
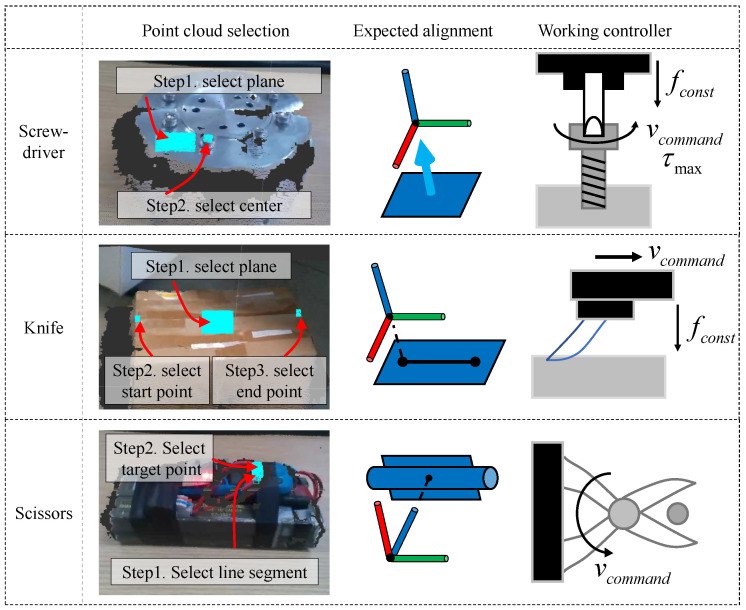
Point cloud selection and working controller of different tools.

Considering a screwdriver tool as an example. A local depth point cloud of the operation area will be reconstructed using multiple frames of RGB-D camera. After the reconstruction, the established point cloud with calibrated color information is displayed on the RViz, and an operator uses the mouse to select the point cloud area of interest for the determination of a working pose of the manipulator end. The selection process varies with a tool carried at the end of the manipulator. When carrying a screwdriver tool, the end-effector needs a normal vector and a center point of the operation plane as shown in Figure 7 expected alignment part of screwdriver. Therefore, in the first step, the plane where a screw hole is located or the vertical plane of the screw axis is selected.

**Figure 8:**
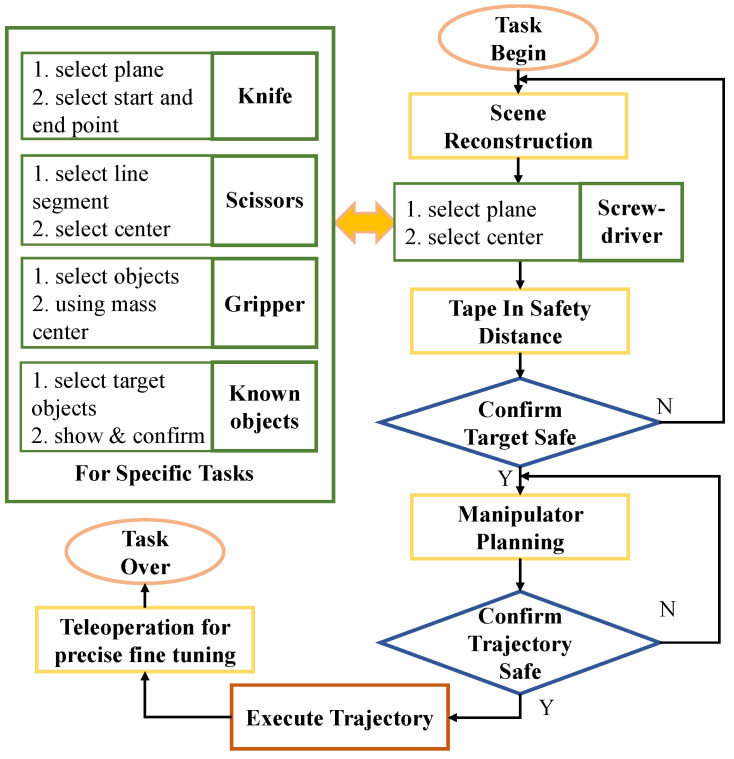
Flowchart of the point cloud-assisted teleoperation operation.

The obtained point cloud data are processed by a plane fitting algorithm to obtain the normal vector. The second step is to select the screw center and then calculate the center of mass of the point cloud to obtain the center point of the operation pose. In the third step, a safety distance is set, which is then used to control the distance of the manipulator in the axial direction to ensure that it will not be far from or too close to the operation target. After the operator completes the operation, the pose of the end-effector can be determined, and it will be displayed on the user interface for the operator to check. If the obtained pose is appropriate, path planning can be performed. The working controller is manually turned on by the operator and can be turned off at any time. The commands from the operator are added into the reference pose of the force controllers. The select plugin of RViz was modified to complete step-by-step selection and confirmation. The trajectory planning process uses the OMPL sampling-based planning method integrated in the Moveit! [26] to avoid obstacles in the surrounding environment efficiently. The point cloud processing methods, such as point cloud reconstruction, normal vector extraction, and line fitting, use algorithms integrated into the Open3d [27] library. The workflow of the operation process is shown in Figure 8.

### 3.2. Force Controllers for Stable Transfer and Tool Operation

Based on the torque-controlled joint and Cartesian impedance control, force controllers for stable transfer and tool operation were designed.

In the EOD transfer tasks, an object to be operated is usually a dangerous explosive. Excessive vibrations can cause the explosives’ properties to change, the switch to trigger, or unexpected release from the gripper. Therefore, this type of task has a high requirement for operational stability where end-effector should exhibit acceleration of less than 2 m/s^2^. An IMU-based end-effector stabilization algorithm is designed, which adopts Cartesian impedance control and IMU attitude measuring to achieve stable transfer and certain vibration suppression.

The force-controlled manipulator can execute the joint torque command on the basis of compensating the gravity. The Cartesian spatial impedance control is implemented in (2), which creates a virtual spring-damping-mass model between current end-effector pose and reference end-effector pose [28].

(2) $$\begin{array}{c} \begin{array}{cc} \tau & =J_{A}^{T}\left(q\right)\left(K\tilde{x}\left(q\right)+D\dot{x}\left(q\right)\right)\\ \tilde{x}\left(q\right) & =f\left(q\right)-x_{d} \end{array} \end{array}$$

where τ is the calculated torque for the joint torque controlled loop. $J_{A}^{T}$ is the analytic Jacobian matrix. K is the stiffness matrix. D is the damping matrix. f(x) is the forward kinematics of the manipulator. x is the cuttent end-effector pose. $x_{d}$ is the reference pose that can be commanded by other controllers. The corresponding system description equation is a second-order dissipation low-pass filter, which can filter high-frequency mechanical vibration signals and attenuate the low-frequency part of the vibration signal, thereby reducing the acceleration overload of explosives.

The IMU is mounted on the torso, and it records the pose of the manipulator base in the gravity coordinate system. Then, the control algorithm in (3) uses the IMU measurement result to compensate for the attitude of the torso by changing the reference frame.

(3) $$\begin{array}{cc} x_{d}\left(k+1\right) & =x_{d}\left(k\right)+\left[v_{d},\omega_{d}\right]\Delta t\\ \left[v_{d},\omega_{d}\right] & =\left[0,\frac{k_{p}}{\Delta t}\left|2acos\left(q_{w}\right)\right|\frac{q_{v}}{\sqrt{1-q_{w}^{2}}}\right]\\ \left[q_{v},q_{w}\right] & =\Delta {}_{ee}^{B}q={}_{ee}^{B}q_{now}^{-1}\cdot {}_{B}^{E}q_{now}^{-1}\cdot \left({}_{B}^{E}q_{0}^{}\cdot {}_{ee}^{B}q_{0}^{}\right) \end{array}$$

where $x_{d}$ is the reference pose sent to controller (2). $\left[q_{v},q_{w}\right]$ is the attitude that the end-effector needs to change expressed as a quaternion. $q_{v}$ is the imaginary part, $q_{w}$ is the real part. $q_{0}$, $q_{\mathrm{now}}$, and Δq are the attitude expressed in quaternion. *E* means Earth frame. *B* means manipulator base frame. ee means manipulator end-effector frame. $k_{p}$ is a controller gain parameter.

The difference between the IMU realtime measurement and the initial measurement is used to obtain the pose compensation of the manipulator end-effector. The desired velocity $\left[v_{d},\omega_{d}\right]$ is obtained through the relationship between the quaternion and the axis angle. Closed-loop feedback control is constructed, and the desired compensation attitude defined by the quaternion is obtained by making a difference with the attitude at the initial moment, which is approximately converted into the angular velocity of the end-effector. Then, the proportional controller is used to convert the error into the speed command of the manipulator end reference frame.

In tool operation tasks, both screwing and cutting need to keep in contact with working object. The contact estimation is hard to acquire in video feedback. Since a screw can fall or rise during the screwing process, and the pitch of the screw is generally an unknown parameter, a force tracking controller of Z-axis in tool frame is designed to help the operator to deal with the contact estimation problem. A constant force is maintained by controller. The force loop adopts the PD control and uses the estimated end-effector force to keep the given target force. The force estimation is achieved by subtracting the gravity term from the filtered joint force sensor readings under the quasistatic assumption [29]. An impedance parameter with a large damping ratio is adopted as an impedance inner loop.

### 3.3. Automatic Tool Replacement Software

**Figure 9:**
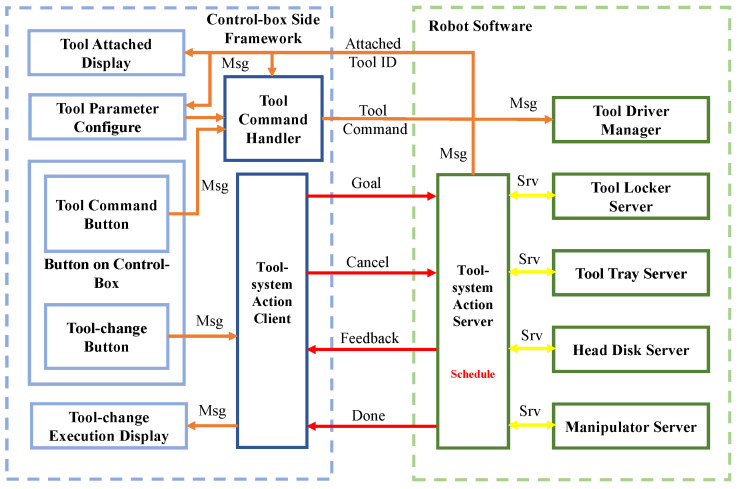
Block diagram of the automatic tool replacement system.

The tool replacement task of the FC-EODR is an autonomous task. An operator only needs to select the corresponding tool according to the task requirements and trigger the button to complete the end tool replacement. The automatic tool replacement software adopts the ROS message service action mode, and its block diagram is shown in Figure 9. The executor uses the service mechanism for triggering and feedback. The overall process uses action management and feeds back the current execution process to the operator. The current tool and continuous tool instructions are delivered by message. The entire tool service system consists of a tool interface module, tool holder trays, screwdriver heads, and manipulator. When replacing tools, a schedule is performed step by step. The manipulator uses movement groups to store movement instructions and employs service to call the movement blocks. Due to various continuous control commands of different tools, the message format is used. Commands for all tools are written uniformly using the message protocol, including information on the position, velocity, torque, and operating mode. In the action procedure, after confirming that the tool is successfully carried, the current tool ID is changed, and the underlying driver starts to exchange data with the tool to ensure that the received tool command can effectively control the carried tool. When the device fails to execute the tool replacement process, the user interface shows the currently executable operation, and then the operator determines the failure state and restores it.

### 3.4. Autonomous Operation Algorithm Based on Visual Perception

When the robot performs the operation tasks on known targets, its operation mode is basically fixed. In this study, an autonomous operation process is designed for the scene of screwing operations, which uses vision and the force tracking control method to realize the screwing operation with screws.

The visual recognition positioning algorithm adopts the previously proposed visual positioning algorithm, the tgcPose6D [30]. The 3D model of the job target is used as an input, and the pose transformation matrix of the target object in the visual coordinate system is obtained. The identified pose is proceeded to the manipulator planner then the camera center is aligned with the screw center. Then, the force controller mentioned in Section 3.2 is turned on, and a constant force of 10 N and a constant screwdriver velocity of about 30 rpm is commanded. The process is completed when the screwdriver is stalled due to reaching the rated torque.

All automated operations are scripted. ROS services are used to complete single process such as calling vision algorithm, turning on force controller, and sending velocity command to tool module. ROS actions are used to perform a sequence of service client and feedback to the operator continuously.

## 4. Experimental Verification

### 4.1. Mobile Platform Passability Test

**Figure 10:**
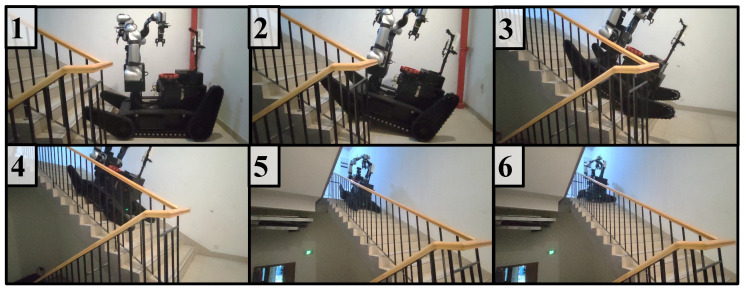
Stair climbing experiment (details of Steps 1–6 are explained in Supplementary Video).

**Figure 11:**
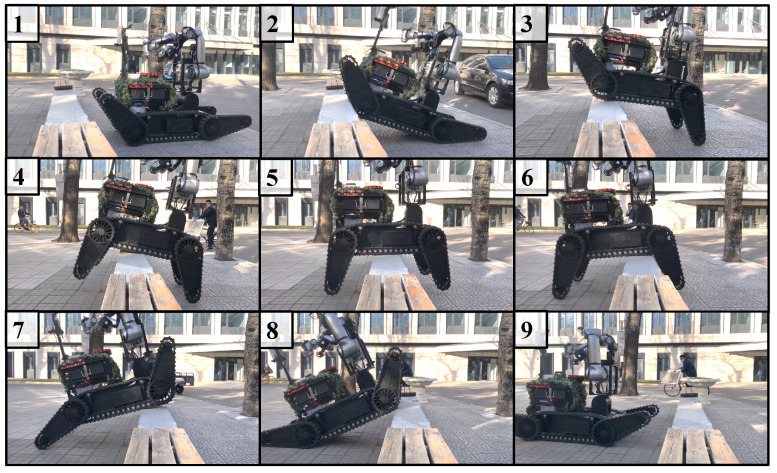
The 0.5-m low wall spanning experiment (details of Steps 1–9 are explained in Supplementary Video).

To verify the passability of the chassis, two experiments were conducted. One experiment included a 30° stair climbing test, which was performed to verify the performance of the track and the power of the drive motor. The results of this experiment are shown in Figure 10. The second experiment was to cross a 0.5-m low wall, and it was conducted to verify the overall robot center of gravity configuration and the drive capability of the swing arm motor. The results of this experiment are shown in Figure 11.

### 4.2. Teleoperation and Autonomous Operation Experiment

**Figure 12:**
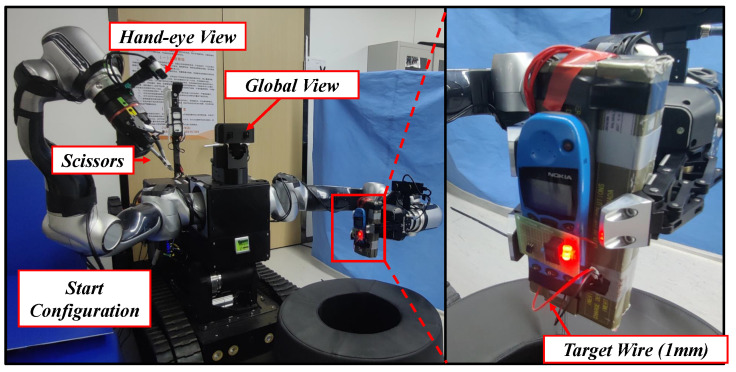
Setup of the teleoperation experiment (see details in Supplementary Video).

For the teleoperation system, the typical scene of wire-cutting during the EOD operation was selected as a research task. The proposed teleoperation method based on the depth point cloud was verified by performing comparative experiments, as shown in Figure 12. In the experiments, the manipulator started from the same configuration. Teleoperation experiments with two mode were conducted. In Mode A, the VR glasses and its controller were used to control manipulator end-effector frame position directly. The stereo camera mounted on left manipulator was used for observation. In Mode B, space-nav mouse and assistance method mentioned in Section 3.1 was used. A hand–eye RGB-D camera and a fisheye camera mounted on the right manipulator as described in Section 2.4 were used for observation. Ten operators were invited, and five successful operations were recorded by each method for each person. Undergraduates and graduate students are included, who have not been exposed to similar robot teleoperation before, but it is not ruled out that they have been exposed to related equipment such as VR games.

Each operator had only one chance in trail to trigger the cut after judging that the wire could be trimmed. The training time, success time, number of attempts, and a comfort interview at the end were recorded. The success rate of the experiment was calculated as a ratio of the number of successes to the number of attempts. The user comfort level is obtained from the questionnaire after the test. The questionnaire contains one question: how do you feel about the overall comfort level during the operation of the robot, very bad, poor, general, good, very good, corresponding to 1–5 point score. User comfort scores are obtained by averaging all ratings. The experimental results are shown in Table 2. The proposed teleoperation method achieved a higher success rate and a shorter average time compared to Mode A. At the same time, the operator’s response to the local remote operation was more labor-saving compared with the global operation, which could easily cause the operator to become tired from maintaining a posture, especially in a long-term operation. For operator training, the global operation required a short training time due to its strong intuition, while the velocity operation required familiarity with the operation of the 6D mouse and the feeling of holding the camera, which consumed much training time. Generally, mature operators were selected to complete the EOD task, so the training time had a slight impact in actual EOD task.

**Figure 13:**
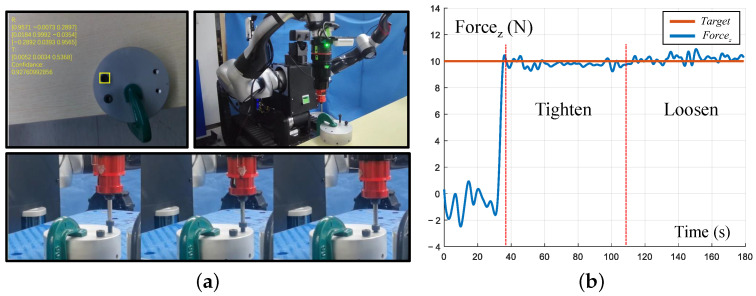
Autonomous operation and end force tracking experiment (see details in Supplementary Video). (**a**) Experiment setup. The workpiece is fixed on the table and screws appear in the field of vision; (**b**) force tracking data in screwing. The contact force is maintained at 10 N in both tighten and loosen procedures.

The screwing operation was selected as an autonomous operation task. First, the tool was replaced automatically. Then, the sensing autonomous operation method presented in Section 3.4 was run to identify and locate the screw. The experimental setup and end force curve during the experiment are shown in Figure 13.

### 4.3. Stable Transfer Capability Test of Suspicious Explosives

**Figure 14:**
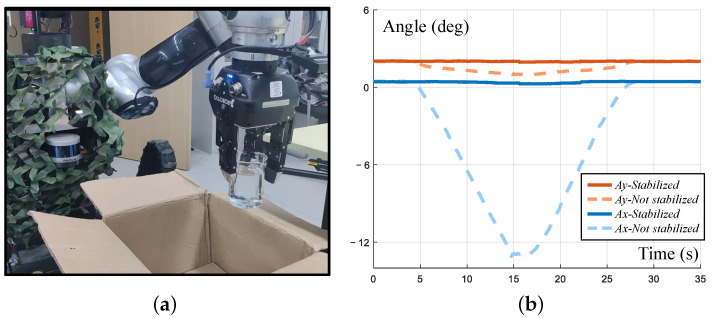
End-effector stabilization experiment. (**a**) Experiment setup. An IMU is mounted on left arm end; (**b**) Experiment result. The fluctuation of the end attitude is slight when the end is stablized.

For the stable transfer task of dangerous explosives or liquids, two IMUs were used to record the pose and acceleration data in the simulated explosive transfer experiment. The end-effector attitude stability and end-effector acceleration decline were compared when the experiment was performed with and without the control algorithm. The end-effector stabilization experiment is shown in Figure 14. The experimental process was to use the front swing arm to simulate the uphill scene. In the closed state, the end-effector would tilt with the inclination of the car body, resulting in the pose transformation relationship of the end-effector in the world coordinate system, as shown by the dashed line in Figure 14b. In Figure 14b, the solid line represents the result when the end-effector tracking control was used and the end-effector remained in the state of the opening moment to keep the direction of gravity stable.

**Figure 15:**
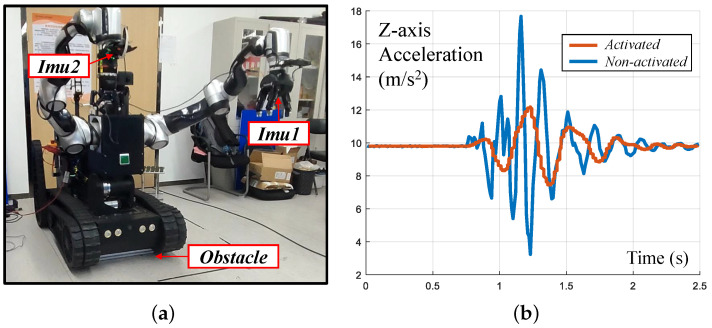
End-effector acceleration decline experiment. (**a**) Experiment setup. Two IMUs are mounted on both arms’end; (**b**) experiment result. About 50% of acceleration is reduced.

The acceleration decline experiment used obstacles to simulate uneven ground. When the chassis touched an obstacle, the IMU at the end of the manipulator detected the acceleration, and the filtering effect was observed by comparing the IMUs of the left and right arms as shown in Figure 15. The first IMU (IMU1) measured the acceleration of the left arm, which turned on the compliance control. The second IMU (IMU2) measured the acceleration of the right arm, which turned on the servo but not the compliance control. For the first contact with an obstacle in 2.5 s, activating the compliance could reduce the acceleration of the end-effector by about 50%. The experimental results are shown in Figure 15b.

### 4.4. Simulated EOD Scenario Experiment

**Figure 16:**
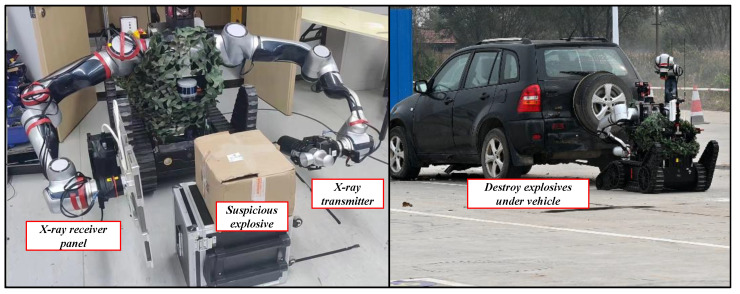
Simulated explosive ordnance disposal scenario experiment (see details in Supplementary Video).

The experimental scene is shown in Figure 16. In this experiment, the ability of the tool interface to carry special equipment was mainly tested. An X-ray machine was used to observe suspicious explosives. In the FC-EODR, an autoreplacement tool module was installed and connected to the receiving plate of the X-ray machine. The left arm grabbed the high-energy X-ray transmitter and aimed it at the object, then transmitter irradiated object through boxes and the X-ray beam hit the imaging plane to form an image. An explosive destroyer is adapted to replacement.

## 5. Conclusions

In this study, a humanoid dual-arm dexterous explosive removal robot system named the FC-EODR is developed. The FC-EODR innovatively introduces force-controlled manipulators, autoreplacement tools, and a high-pass mobile platform. The FC-EODR includes teleoperation with the point cloud perception and autonomous tool replacement system and can realize autonomous visual operation and stable transfer control in dangerous explosive scenes. The passability through complex terrains, including stairs and low walls, and performance in immersive remote operations and autonomous visual operations of the proposed FC-EODR are verified by experiments. The results show that the proposed robot system can achieve good performance in dexterous explosive removal operations and can replace humans in hazardous removal operations.

In future research, the autonomy and intelligence of operation will be further analyzed, and more human experience will be used in the operation of EOD robots to improve their accuracy, flexibility, and intelligence.

## Figures and Tables

**Table 1:** Performance parameters of the manipulator.

Items	Performance Parameters
Payload	10 kg
Workspace Radius	914 mm
Degree of Freedom	7
Weight	26 kg
Repeatability	±0.03 mm
Force Control Relative Accuracy	0.2 N, 0.1 Nm
Joint1 Range	[−179°, 179°]
Joint2 Range	[−90°, 90°]
Joint3 Range	[−179°, 179°]
Joint4 Range	[0°, 179°]
Joint5,6,7 Range	[−179°, 179°]

**Table 2:** Results of the Teleoperation Comparison Experiment.

Parameter	Mode A	Mode B
Average success time (s)	107.9 ± 65.3	70.1 ± 34.1
Success rate	0.46	0.71
Average learn time (min)	8.6 ± 4.1	15.9 ± 9.2
User comfort score	3.3 ± 0.82	4.1 ± 0.56

## Data Availability

Not applicable.

## Supplementary Materials

The following supporting information can be downloaded at: https://www.mdpi.com/article/10.3390/biomimetics8010067/s1, Video S1: FC-EODR performance verification.
